# Supplementary material for: STARTER: a stand-alone reconfigurable and translational organ-on-chip platform based on modularity and open design principles
Source: Lab Chip. 2026 Jan 26;26(3):604–17. doi: 10.1039/d5lc00756a (PMC12834090; doi:10.1039/d5lc00756a)
Supplement: LC-026-D5LC00756A-s001 [file LC-026-D5LC00756A-s001.pdf]

Supplementary Information (SI) for Lab on a Chip.
This journal is © The Royal Society of Chemistry 2026

Supplementary Information

STARTER : A Stand-Alone Reconfigurable and Translational Organ-on-Chip Platform based on Modularity and Open Design Principles

Aniruddha Paul*[1], Eric R. Safai*[2], Laura E. de Heus[2], Anke R. Vollertsen[2], Kevin Weijgertse[3], Bjorn de Wagenaar[3], Hossein E. Amirabadi[4], Evita van de Steeg[3], Mathieu Odijk[1], Andries D. van der Meer[2], Joshua Loessberg-Zahl[1,2]

[1] BIOS Lab on Chip Group, Mesa+ Institute of Nanotechnology, University of Twente, The Netherlands

[2] Department of Bioengineering Technologies, University of Twente, The Netherlands

[3] Netherlands Organization for Applied Scientific Research (TNO), Leiden, The Netherlands

[4] AZAR Innovations, Utrecht, The Netherlands

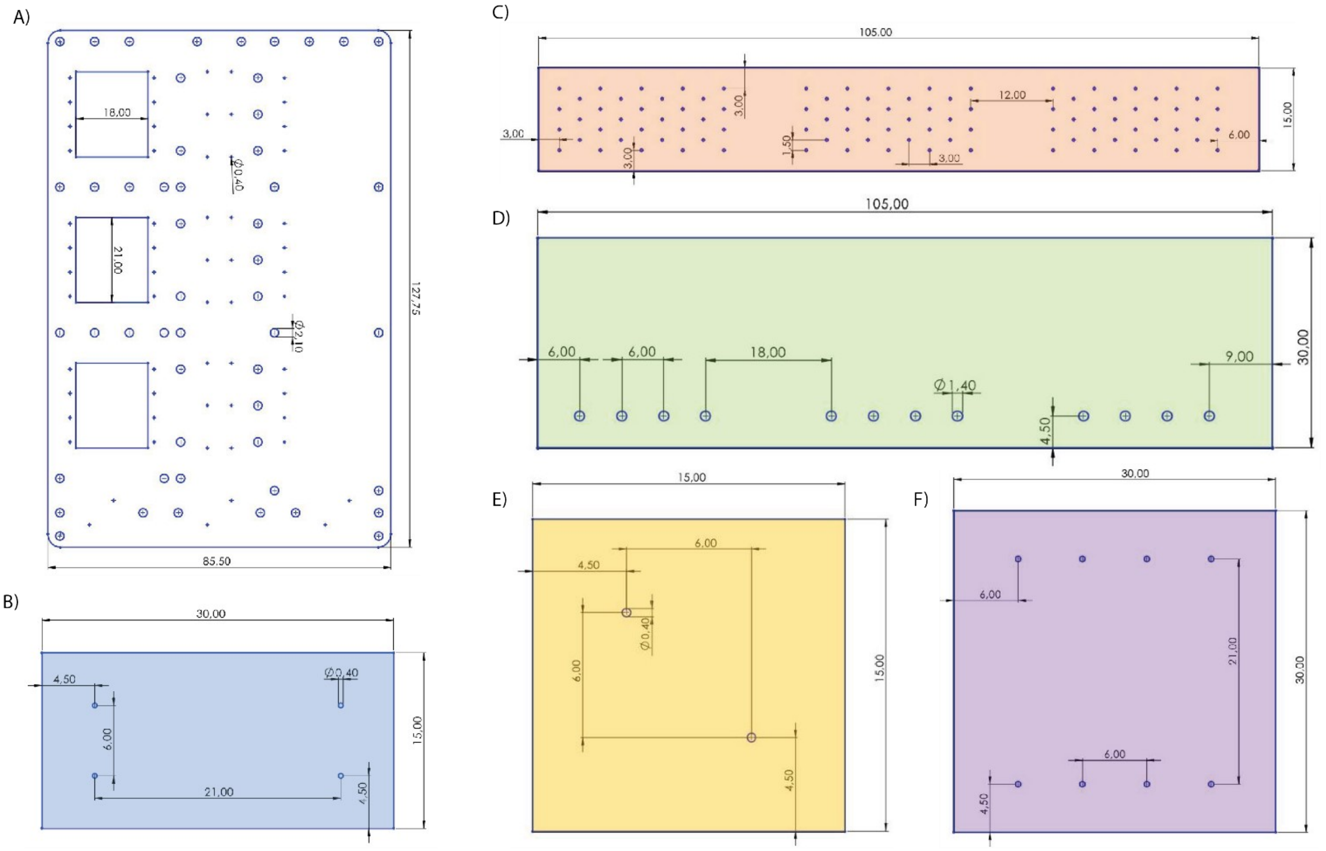

Figure S1 – a) Schematic of FCB. b) Reservoir Block schematic. c) Routing Block schematic. d) Pump Block schematic. e) Sensor Block schematic. f) Organ-on-Chip schematic

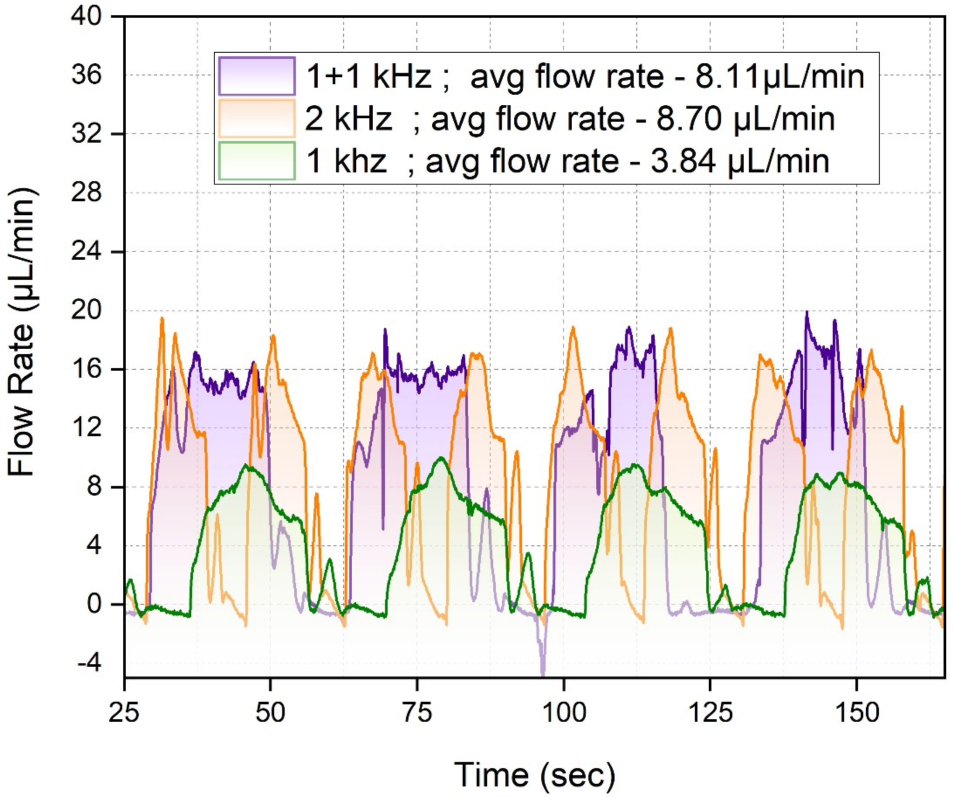

Figure S2 – Flow rate variation comparison between isolated pump operations at 1kHz and 2kHz and combined operation of two pumps running at 1kHz.

Step 1 – OoC cell seeding. OoCs are seeded off platform till maturation.

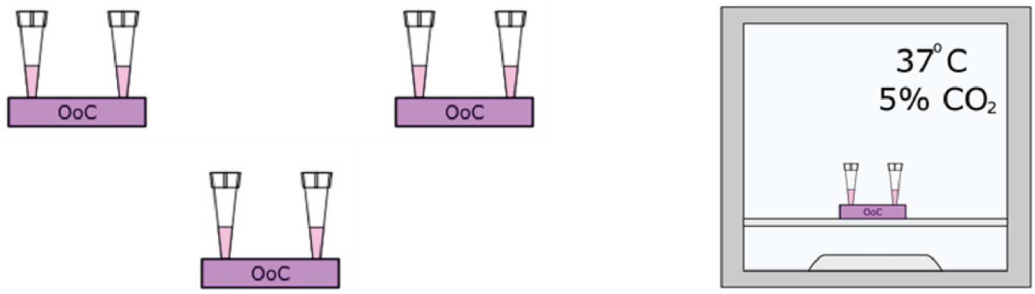

Step 2 – Sterilizing and priming STARTER. Dummy priming chips are connected in plaace of OoCs. The platform is sterilized by flushing with 70% ethanol for 30 min followed by flushing phosphate buffered saline(PBS) for 30 min in the incubator.

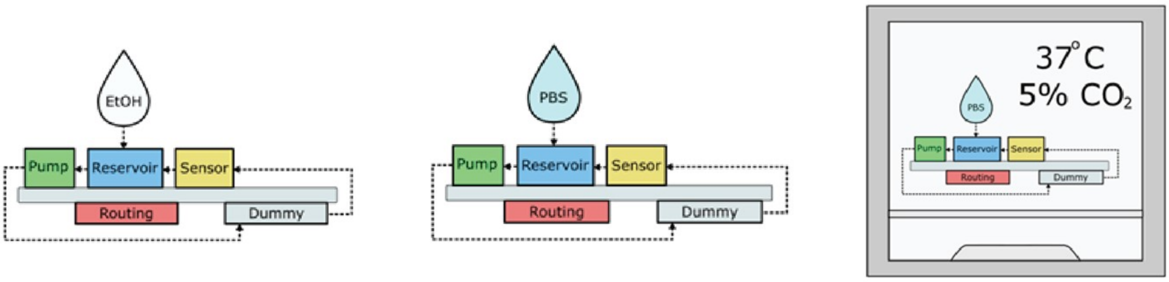

Step 3 – OoC integration on STARTER. STARTER is flushed with acclimatized media. The dummy priming chips are then swapped with previously cultured OoCs. The OoCs on STARTER are recirculated with media for required days in incubator.

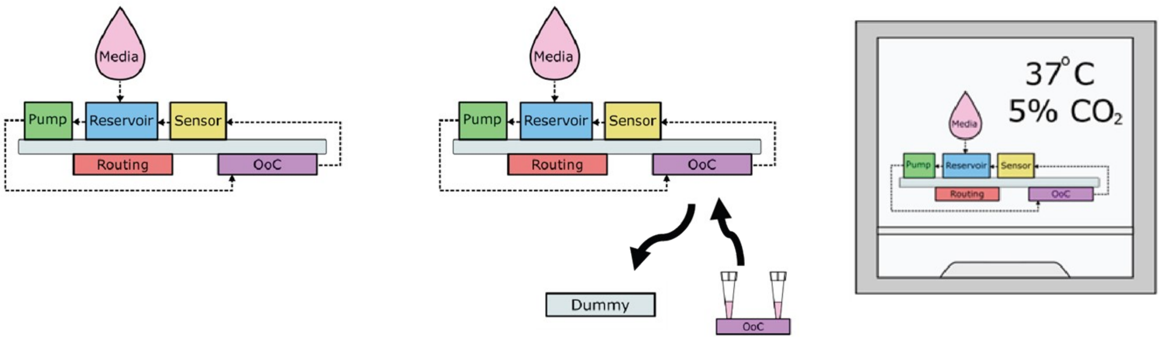

Figure S3 – The priming and OoC integration steps for STARTER.

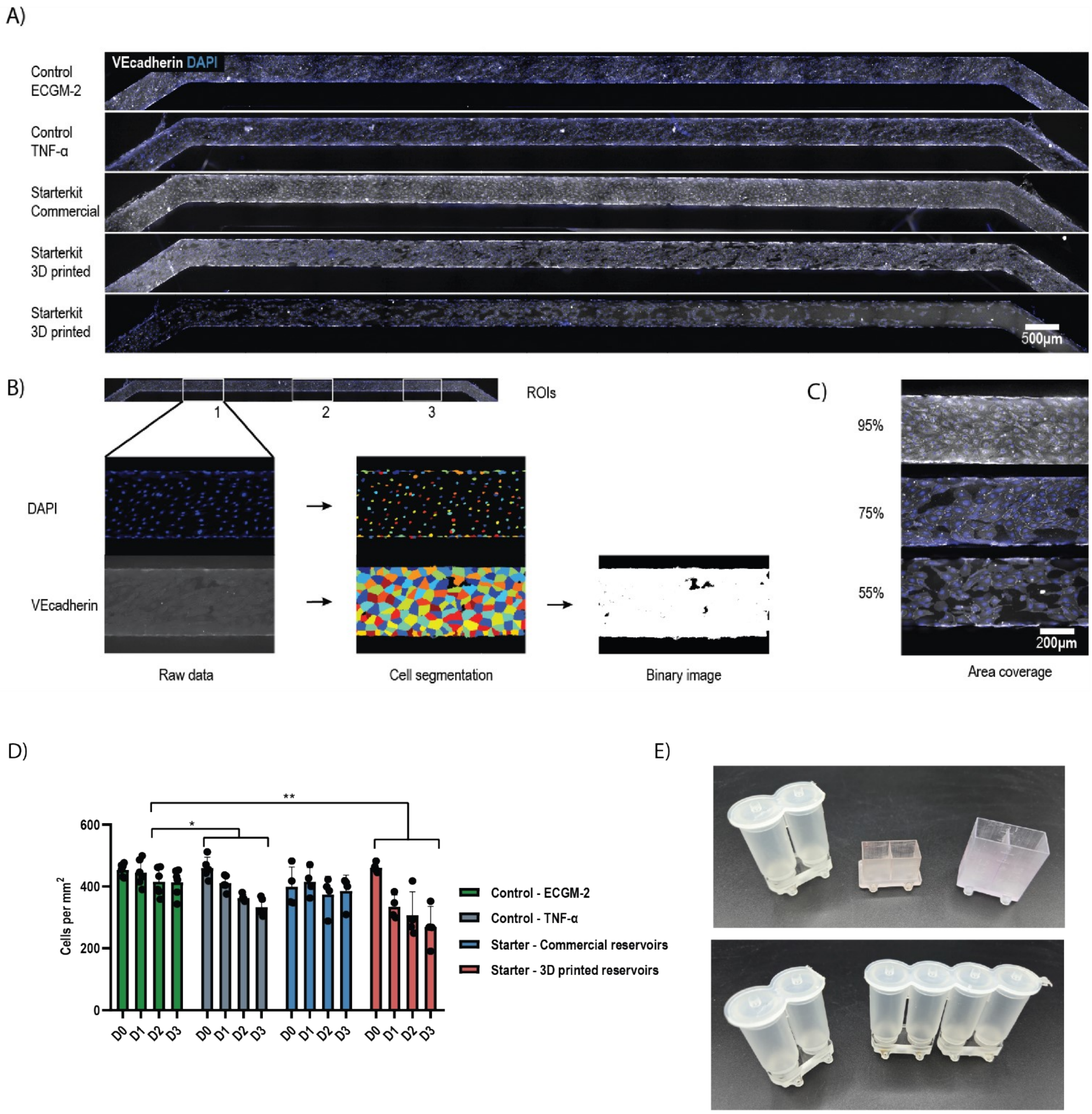

Figure S4 – a) Area coverage of HUVECs after 3 days of cell culture in different conditions. b) Analysis of area covered by cells. c) Example of 95%, 75% and 55% area coverage. d) Cell number during 3 days of in vitro cell culture of HUVECs in a vessel-on-chip (VoC) in regular medium (ECGM-2). Decline in cell numbers in STARTER with 3D printed reservoirs is comparable with pro-inflammatory stimulus with 5ng/ml TNF-α. e) Images of Commercial and 3D printed reservoirs.

*Medium refreshment was either performed on a rocker platform or continuously for control and STARTER conditions, respectively.

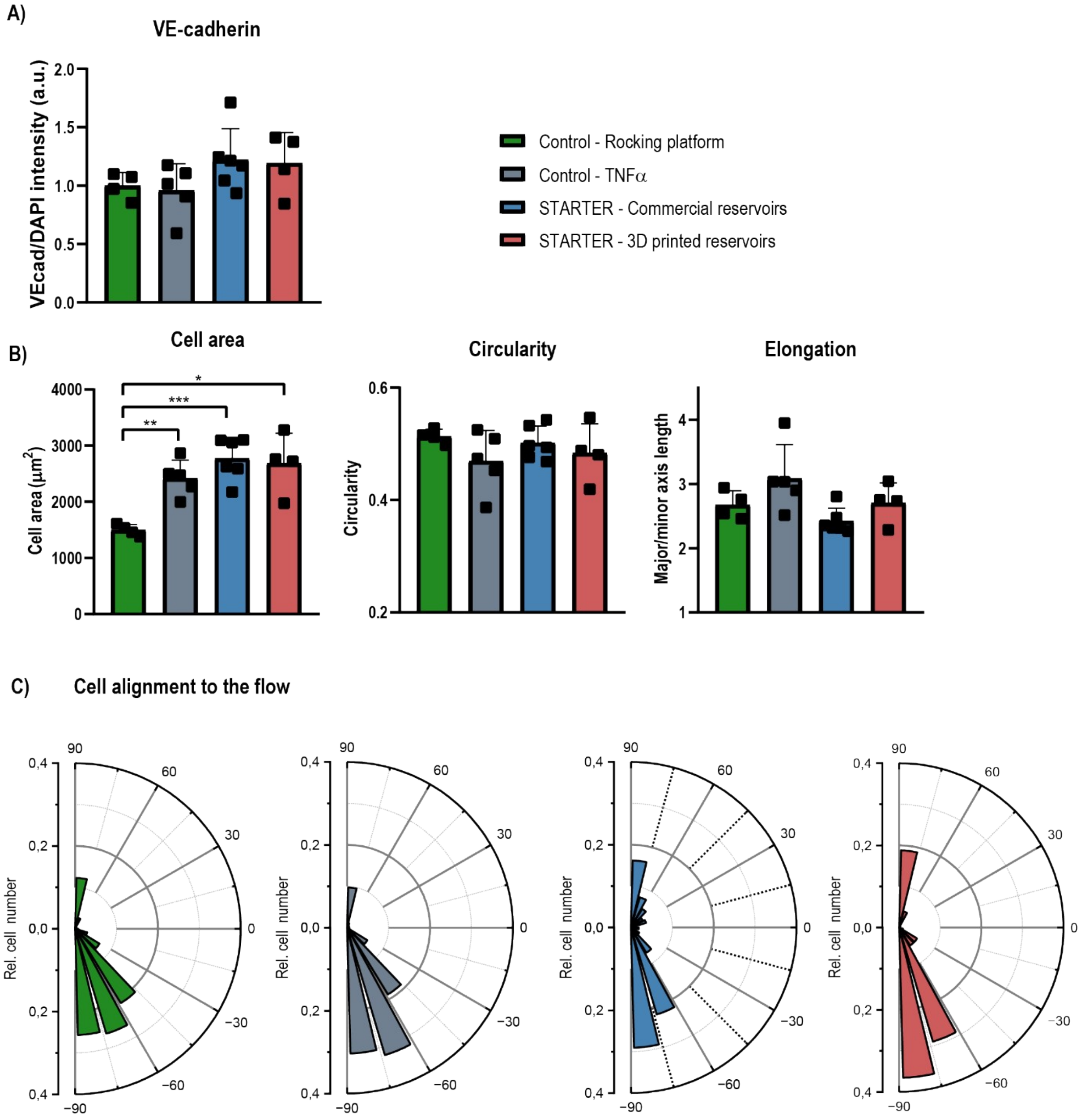

Figure S5 – a) Comparison of total VE-cadherin intensity normalized to the total DAPI intensity. b) General Cell morphology Comparison of cell area, circularity and cell elongation. c) Comparsion of Cell orientation with respect to direction of flow.

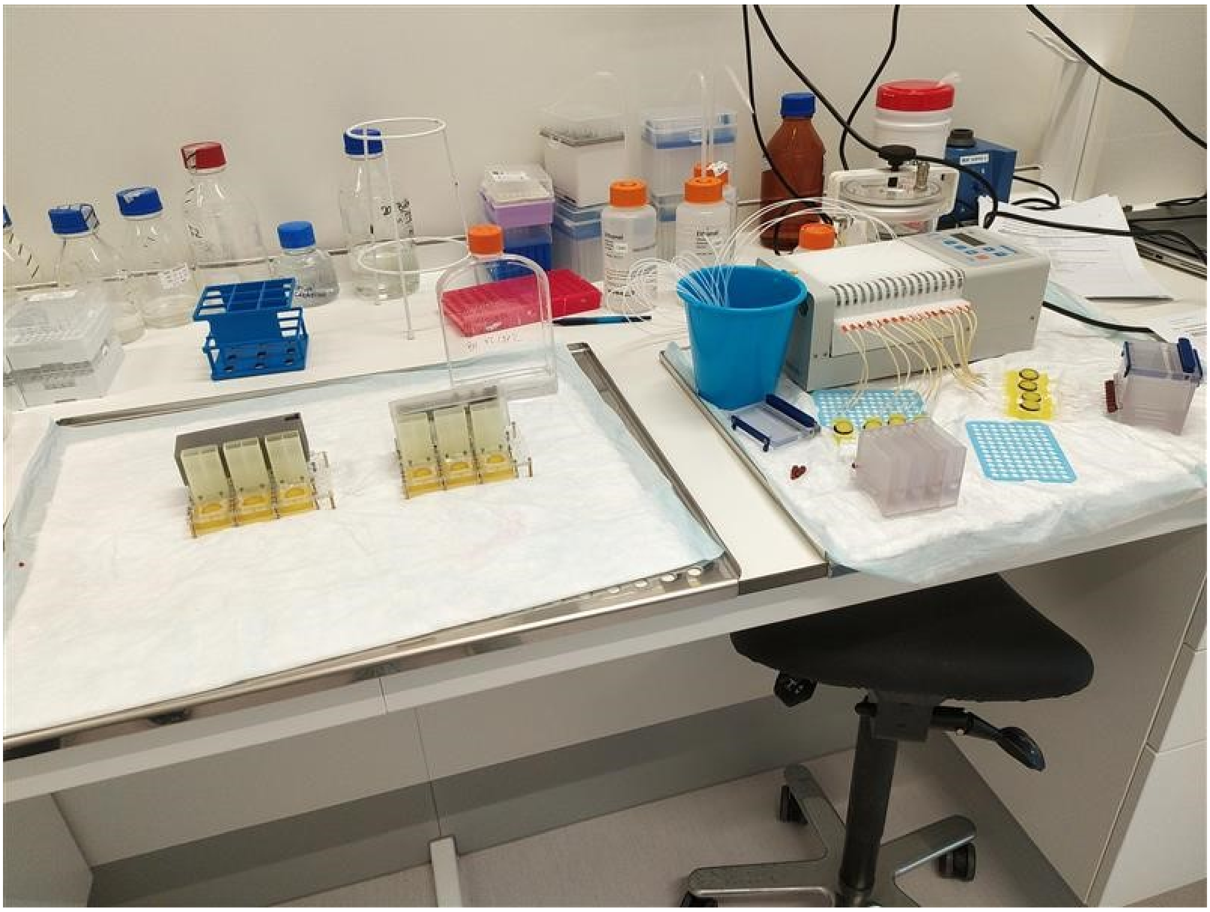

Figure S6 – Comparison of workflows of a traditional experimental setup for 8 IEBCs (right) and 6 IEBCs on STARTER platforms (left).

Supplementary Videos-

S1 - Filling of STARTER in Combined Operation

S2 – Mixing and Gradient Generation
